# Prevalence, risk factors, and clinical characteristics of pulmonary embolism in patients with acute exacerbation of COPD in Plateau regions: a prospective cohort study

**DOI:** 10.1186/s12890-024-02915-z

**Published:** 2024-02-27

**Authors:** Chenlu Yang, Yajun Tuo, Xuefeng Shi, Jie Duo, Xin Liu, Fang Zhang, Xiaokai Feng

**Affiliations:** 1 Department of Respiratory and Critical Care Medicine, Qinghai Provincial People’s Hospital, Qinghai, China; 2 Department of Epidemiology and Biostatistics, School of Basic Medicine, Institute of Basic Medical Sciences Chinese Academy of Medical Sciences, Peking Union Medical College, Beijing, China; 3 Department of Respiratory and Critical Care Medicine, Beijing Institute of Respiratory Medicine, Beijing Chao-Yang Hospital, Capital Medical University, Beijing, China; 4 Institute of Disaster and Emergency Medicine, Tianjin University, Tianjin, China

**Keywords:** Acute exacerbations of chronic obstructive pulmonary disease, Pulmonary thromboembolism, Plateau regions, Prevalence, Risk factors

## Abstract

**Background and objective:**

To investigate pulmonary thromboembolism (PE) in acute exacerbation of chronic obstructive pulmonary disease (AE-COPD) patients in plateau regions, we performed a prospective cohort study to evaluate the prevalence, risk factors and clinical characteristics of PE in the cohort of hospitalized patients at high altitude.

**Methods:**

We did a prospective study with a total of 636 AE-COPD patients in plateau regions. Demographic and clinical data, laboratory data, including ultrasound scans of the lower extremities and cardiac ultrasound, and computed tomographic pulmonary angiography (CTPA) variables were obtained, and comparisons were made between groups with and without PE. We also conducted logistic regression to explore the risk factors of PE.

**Results:**

Of the 636 patients hospitalized with AE-COPD (age 67.0 ± 10.7 years, 445[70.0%] male), 188 patients developed PE (29.6% [95% CI: 26.0%, 33.1%]). Multivariable logistic regression showed that ethnic minorities, D-dimer > 1 mg/L, AST > 40 U/L, chest pain, cardiac insufficiency or respiratory failure, Padua score > 3, and DVT were associated with a higher probability of PE.

**Conclusions:**

The prevalence of PE is high and those with a higher Padua score, the occurrence of deep venous thrombosis, higher neutrophil count, chest pain, cardiac insufficiency or respiratory failure, higher levels of AST, and a higher level of D-dimer had a higher risk of PE. The analysis of AE-COPD may help to provide more accurate screening for PE and improve clinical outcomes of patients with AE-COPD in plateau regions.

**Supplementary Information:**

The online version contains supplementary material available at 10.1186/s12890-024-02915-z.

## Introduction

From 1990 to 2015, the occurrence of chronic obstructive pulmonary disease (COPD) rose by 44.2%, impacting 174.483 million individuals, with a concurrent 11.6% increase in the mortality rate [1]. Due to its high incidence, mortality, and medical costs [2–4], COPD places a significant socioeconomic burden globally, emerging as a pivotal public health concern. Acute exacerbation of COPD (AE-COPD) is characterized by a sudden escalation of symptoms beyond the typical daily fluctuations, necessitating additional therapeutic intervention [5]. A prethrombotic condition is associated with AE-COPD [6]. The additional factors like immobility and infection, in conjunction with AE-COPD, heightens the susceptibility to venous thromboembolism (VTE) among hospitalized patients. Patients with COPD may experience exacerbated gas exchange and hypoxia when exposed to low barometric pressure and high-altitude hypoxic environments, which further increases the risk of pulmonary hypertension and cor pulmonale, ultimately contributing to increased mortality [7]. In addition, the prevalence of pulmonary embolism (PE) has been noted to be higher among AE-COPD patients [8]. Additionally, COPD is a significant risk factor for PE. Yet, the similarity in clinical symptoms between PE and AE-COPD complicates the diagnosis of PE in individuals experiencing AE-COPD. Delayed anticoagulation therapy by clinicians contributes to a poorer prognosis. Postmortem findings additionally indicated that the incidence of PE in individuals with COPD ranged from approximately 28–51% [9].

High altitude exposure constitutes to thromboembolic disorders [10–13], including venous thrombosis [14–17], pulmonary thromboembolism, mesenteric vein thrombosis, cerebral vein thrombosis, and deep vein thrombosis (DVT) [10, 18–20], primarily attributed to blood hypercoagulability. An earlier study indicated that people living in high-altitude areas for one year experienced thromboembolic events (including DVT and PE) 30 times more than those living in low altitude areas [13]. Compare to regions at lower altitudes, prolonged exposure to high altitudes is associated with an elevated risk of stroke and the subsequent need for hospitalization (13.7 versus 1.05 in 1000 people) [20]. Furthermore, based on a 5-year retrospective cohort study of the US military academies, the risk of thromboembolism in areas with higher elevations (2210 m) is twice that of sea level [21].

Thereby, we aimed to explore the prevalence, risk factors, and clinical characteristics of PE in plateau regions by performing a prospective cohort study among in-hospital patients with confirmed AE-COPD.

## Methods

### Study population

We prospectively included all consecutive AE-COPD inpatients (*n* = 1,042) defined as the exacerbation of respiratory symptoms (dyspnea, cough, sputum, fever) in COPD patients, exceeding daily standards and requiring changes in medication treatment plans [5], which were previously diagnosed as COPD according to the global initiative for obstructive lung disease (GOLD) criteria [22], from January 1, 2019, to October 31, 2021, in Qinghai Provincial People’s Hospital. The study was approved by the Research Ethics Board at Qinghai Provincial People’s Hospital, Qinghai University (Ethical number:2018-53) and was in accordance with the Helsinki Declaration. Oral consent was obtained from patients involved before enrollment.

**Fig. 1:**
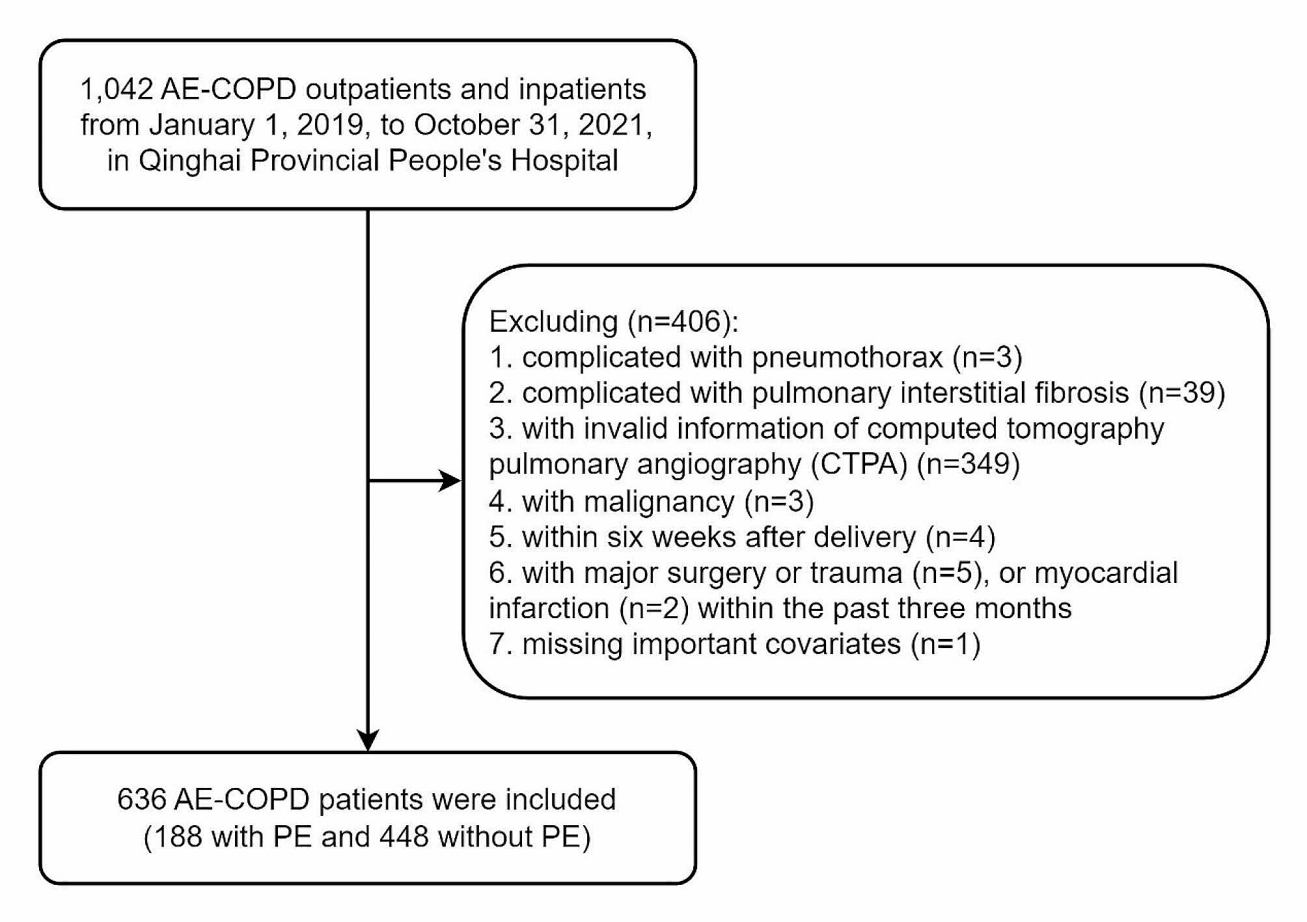
Flow-chart of this study

Furthermore, we excluded those: (1) complicated with pneumothorax (*n* = 3); (2) complicated with pulmonary interstitial fibrosis (*n* = 39); (3) with invalid information of computed tomography pulmonary angiography (CTPA) (*n* = 349); (4) with malignancy (*n* = 3); (5) within six weeks after delivery (*n* = 4); (6) with major surgery or trauma (*n* = 5), or myocardial infarction (*n* = 2) within the past three months; (7) missing important covariates (*n* = 1). Finally, this study enrolled 636 AE-COPD patients (Fig. 1).

### PE diagnosis

Within the first 24 h of admission, Chest CT angiography (CTA) was conducted using a 16-section multi-detector CT scanner (GE Light Speed 16; GE Healthcare, Milwaukee, Wisconsin, USA). Patients received a 100 mL injection of non-ionic contrast media (Iohexol Omnipaque 300/100; GE Healthcare, Milwaukee, WI, USA) through an 18G needle in the antecubital vein, administered at a rate of 4 s using a power injector (Medrad Stellant Dual; Medrad, Indianola, PA, USA). A dedicated workstation (Advanced Workstation 4.0; GE Healthcare) was employed for the execution of Chest CTA. The confirmation of pulmonary embolism (PE) was achieved upon the identification of an intraluminal filling defect, enveloped by intravascular contrast, or the observation of complete occlusion within the pulmonary arterial lumen at any location throughout the pulmonary arteries.

As described in an earlier study [23], PE was identified on CT scans by the presence of a clearly defined filling defect within the pulmonary artery, which was distinctly outlined and observed in a minimum of two consecutive image sections, positioned either centrally within the vessel or displaying acute angles at its interface with the vessel wall. It was diagnosed as DVT according to a low-attenuating partial or complete intraluminal filling defect surrounded by a high-attenuating ring of enhanced blood which was identified at least two consecutive transverse images [24]. Proximal DVT was characterized by thrombosis at or above the popliteal vein level, while distal DVT was identified by thrombosis affecting the axial calf veins.

### Covariates collection

We collected the social demography characteristics, disease history, disease characteristics, symptoms, comorbidities, preventive treatment, physical examination, laboratory examination, electrocardiogram and echocardiography, and treatment of ventilation as well as peripherally inserted central catheter among AE-COPD patients based on electronic medical records and diagnosis and treatment processes. Body mass index (BMI) was calculated as weight (kg) divided by the square of height (m).

### Data analysis

We used the Shapiro-Wilk test to assess the normality of the continuous variables. We described those with normal distribution as means ± standard deviations and compared them by using the independent students’ *t*-test, and those with skew distribution were described as medians (P25, P75) and compared by the nonparametric Wilcoxon test. Categorical variables were shown as n (%) and compared by using the χ^*2*^ test and the Fisher exact probability test. We calculated the odds ratio (OR) and 95% CI for the risk factors of PE through multivariable logistic regression. Independent variables were selected based on statistical significance combined with professional knowledge in univariable analysis while considering the correlation between variables to avoid overfitting. The final model was determined based on the Akaike information criterion. The dose-response relationship between continuous variables and the logit-transformed PE probability was constructed through the restricted cubic spline function (Figure S1), and the variables with nonlinear correlation were converted into categorical variables when conducting multivariable logistic regression. The variables beyond the specified category and those exceeding the median ± three times the quartile interval were defined as outliers. Continuous variables with missing values were imputed by using multiple imputation methods based on Monte Carlo simulation and categorical variables were imputed through conditional imputation methods based on mode. We used a two-tailed test, and *P* < 0.05 was considered statistically significant. We used SAS 9.4 (SAS Institute, Cary, NC, USA) and R (version 4.2.1, https://www.r-project.org/) for all statistical analyses.

## Results

### Prevalence of PE and characteristics of AE-COPD patients

We enrolled 636 AE-COPD patients with a male proportion of 70% and an average age of 67.0 ± 10.7 years. Among them, 188 patients had PE, and the prevalence of PE was 29.6% (95% CI: 26.0%, 33.1%). Compared to non-PE patients, the proportion of PE patients with a Padua score ≥ 4 was higher, while the proportion of Han nationality was lower. The proportion of AE-COPD patients with PE who had a history of hypertension was lower, and the proportion of strokes within the past 3 months and preventive treatment was higher. Moreover, patients with PE had a higher proportion of chest pain, cardiac insufficiency or respiratory failure, and DVT (Table 1).

**Table 1:** Sociodemographic and disease characteristics of patients with AE-COPD

Variables	All (*n* = 636)	Non-PE (*n* = 448)	PE (*n* = 188)	*P*-value
**Sociodemographic characteristics**				
Males	445 (70.0)	308 (68.8)	137 (72.9)	0.301
Age, years	67.0 ± 10.7	66.7 ± 10.5	67.7 ± 11.2	0.283
Han nationality	434 (68.2)	326 (72.8)	108 (57.4)	**< 0.001**
BMI, kg/m^2^	23.5 ± 4.9	23.7 ± 4.9	23.0 ± 4.9	0.140
Smoked	226 (35.5)	157 (35.0)	69 (36.7)	0.690
**Disease characteristics**				
Bed time > 3 days	75 (11.8)	51 (11.4)	24 (12.8)	0.622
Padua score ≥ 4	224 (35.2)	124 (27.7)	100 (53.2)	**< 0.001**
Geneva score ≥ 3	122 (19.2)	82 (18.3)	40 (21.3)	0.385
**Disease history**				
Hypertension	285 (44.8)	219 (48.9)	66 (35.1)	**0.001**
Diabetes	50 (7.9)	41 (9.2)	9 (4.8)	0.062
Autoimmune disease	2 (0.3)	2 (0.4)	0 (0.0)	0.359
Stroke within 3 months	23 (3.6)	11 (2.5)	12 (6.4)	**0.016**
Ventilation within 3 months	60 (9.4)	48 (10.7)	12 (6.4)	0.088
VTE	22 (3.5)	14 (3.1)	8 (4.3)	0.477
**Preventive treatment**				
LMWH therapy	242 (38.1)	140 (31.3)	102 (54.3)	**< 0.001**
New anticoagulant	40 (6.3)	18 (4.02)	22 (11.7)	**< 0.001**
VTE prophylaxis	289 (45.4)	174 (38.8)	115 (61.2)	**< 0.001**
**Symptoms**				
Dyspnea	236 (37.1)	173 (38.6)	63 (33.5)	0.224
Chest pain	100 (15.7)	56 (12.5)	44 (23.4)	**0.001**
Hemoptysis	17 (2.7)	12 (2.7)	5 (2.7)	0.989
Palpitation	27 (4.2)	17 (3.8)	10 (5.3)	0.384
Syncope	7 (1.1)	4 (0.9)	3 (1.6)	0.438
Lower limb edema	353 (55.5)	239 (53.3)	114 (60.6)	0.091
Lower limb pain	11 (1.7)	5 (1.1)	6 (3.2)	0.067
**Comorbidities**				
Infection	535 (84.1)	382 (85.3)	153 (81.4)	0.221
Cardiac insufficiency or respiratory failure	422 (66.4)	350 (78.1)	72 (38.3)	**< 0.001**
DVT	39 (6.1)	5 (1.1)	34 (18.1)	**< 0.001**
Proximal DVT	19 (3.0)	0 (0.0)	19 (10.1)	**< 0.001**
Distal DVT	15 (2.4)	4 (0.9)	11 (5.9)	**< 0.001**
Intermuscular DVT	9 (1.4)	1 (0.2)	8 (4.3)	**< 0.001**
Cardiac injury	23 (3.6)	12 (2.7)	11 (5.9)	0.051
Acute renal insufficiency	5 (0.8)	4 (0.9)	1 (0.5)	0.638
Hepatic insufficiency	20 (3.1)	13 (2.9)	7 (3.7)	0.588
Tricuspid regurgitation	496 (78.0)	354 (79.0)	142 (75.5)	0.333
Pericardial effusion	152 (23.9)	103 (23.0)	49 (26.1)	0.407
Pulmonary hypertension	489 (76.9)	349 (77.9)	140 (74.5)	0.349

Abbreviation: AECOPD: Acute Exacerbation of Chronic Obstructive Pulmonary Disease; BMI: Body Mass Index; DVT: Deep Venous Thrombosis; VTE: Venous Thromboembolism; LMWH: Low Molecular Weight Heparin Therapy

The results of physical and laboratory examination suggested that the neutrophil count, D-dimer, alanine transaminase, aspartate aminotransferase, and lactate dehydrogenase were higher in patients with PE, while systolic blood pressure, albumin, and proportion of right branch block was lower. There was no significant difference between the two groups in terms of ventilation and peripherally inserted central catheter treatment (Table 2).

**Table 2:** Physical examination, laboratory test indexes, and treatment in patients with AE-COPD

Variables	All (*n* = 636)	Non-PE (*n* = 448)	PE (*n* = 188)	*P*-value
**Physical examination**				
Systolic blood pressure, mmHg	126.2 ± 19.1	127.2 ± 19.3	123.9 ± 18.5	**0.050**
Diastolic blood pressure, mmHg	78.6 ± 13.6	79.1 ± 14.1	77.4 ± 12.3	0.146
Heart rate, /min	84 (75, 95)	84 (75, 95)	84 (76, 98)	0.365
Respiratory rate, /min	20 (19, 22)	20 (19, 22)	20 (19, 22)	0.832
**Laboratory test**				
White blood cell count, ×10^9^	5.6 (4.5, 7.0)	5.5 (4.5, 6.8)	5.9 (4.5, 7.9)	0.064
Neutrophil count,×10^9^	3.8 (2.9, 5.2)	3.7 (2.8, 5.1)	4.1 (3.0, 6.2)	**0.011**
Hemoglobin, g/L	175 (150, 201)	174 (152, 203)	177 (147, 198)	0.551
Platelet count, ×10^9^	134 (98, 175)	137 (101, 176)	129 (91, 172)	0.059
Erythrocyte sedimentation rate, mm/h	2.0 (1.0, 7.0)	2.0 (1.0, 6.6)	2.0 (1.0, 8.2)	0.395
D-dimer, mg/L	1.4 (0.9, 3.0)	1.3 (0.9, 2.4)	2.1 (1.2, 5.3)	**< 0.001**
PT, s	12.9 (11.9, 14.3)	12.8 (11.9, 14.3)	13.1 (11.9, 14.3)	0.480
APTT, s	32.0 (28.4, 36.8)	32.0 (28.6, 36.8)	32.0 (27.7, 36.8)	0.671
OI, mmHg	270 (220, 330)	274 (220, 330)	257 (218, 316)	0.111
Total protein, g/L	61.3 (56.8, 66.1)	61.5 (56.9, 66.0)	60.70 (55.80, 66.40)	0.602
Albumin, g/L	34.7 (31.3, 38.0)	34.8 (31.6, 38.2)	34.3 (30.5, 37.3)	**0.040**
ALT, U/L	18 (12, 30)	18 (12, 27)	22 (14, 44)	**< 0.001**
AST, U/L	24 (19, 35)	23 (19, 32)	26 (20, 43)	**0.002**
LDH, IU/L	265.0 (207.5, 340.0)	258.0 (204.0, 332.0)	275.0 (228.2, 360.0)	**0.010**
BUN, mmol/L	7.2 (5.1, 9.7)	6.9 (5.0, 9.6)	7.6 (5.2, 9.9)	0.183
Uric acid, µmol/L	425.0 (326.5, 560.5)	424.5 (325.5, 559.0)	434.5 (328.0, 568.0)	0.724
Creatinine, µmol/L	75.0 (63.0, 89.0)	75.0 (62.0, 88.2)	76.5 (64.0, 90.0)	0.745
cTnI, ng/L	18.1 (6.3, 50.6)	16.2 (6.1, 49.9)	22.1 (7.4, 53.6)	0.298
CK-MB, U/L	13.0 (10.0, 18.3)	13.0 (10.0, 18.6)	13.0 (9.5, 18.0)	0.679
NT-proBNP, pg/mL	410.0 (119.0, 924.4)	406.0 (124.5, 910.2)	425.4 (105.0, 975.1)	0.956
**Electrocardiogram and echocardiography**				
Sinus tachycardia	43 (6.8)	35 (7.8)	8 (4.3)	0.103
Nonspecific T-wave inversion	21 (3.3)	12 (2.7)	9 (4.8)	0.175
Poor R-wave progression	57 (9.0)	35 (7.8)	22 (11.7)	0.117
Right bundle branch block	104 (16.4)	83 (18.5)	21 (11.2)	**0.022**
S1Q3T3 pattern	35 (5.5)	25 (5.6)	10 (5.3)	0.895
LA diameter, mm	36 (33, 41)	36 (33, 41)	36 (32, 41)	0.963
LVESVI, mL/m^2^	45 (41, 48)	45 (41, 48)	45 (41, 48)	0.588
LVEDVI, mL/m^2^	28 (25, 31)	28 (25, 31)	28 (25, 31)	0.454
Simpson biplane EF, %	65.0 (60.0, 69.0)	65.0 (60.9, 68.5)	65.0 (60.0, 70.0)	0.723
RA diameter, mm	44 (38, 50)	44 (38, 50)	43 (38, 49)	0.266
RV diameter, mm	36 (30, 39)	36 (30, 40)	36 (31, 39)	0.876
PA diameter, mm	29 (25, 32)	29 (25, 32)	28 (24, 32)	0.108
PASP, mmHg	63 (48, 82)	65 (49, 83)	60 (47, 81)	0.292
**Treatment**				
Ventilation	57 (9.0)	39 (8.7)	18 (9.6)	0.726
PICC	6 (0.9)	5 (1.1)	1 (0.5)	0.487

Abbreviation: AECOPD: Acute Exacerbation of Chronic Obstructive Pulmonary Disease; PT: Prothrombin Time; APTT: Activated Partial Thromboplastin Time; OI: Oxygenation Index; ALT: Alanine Transaminase; AST: Aspartate Aminotransferase; LDH: Lactate Dehydrogenase; BUN: Blood Urea Nitrogen; cTnI: Cardiac Troponin I; CK-MB: Creatine Kinase-MB; DVT: Deep Vein Thrombosis; NT-proBNP: N-terminal Prohormone B-type Natriuretic Peptide; LA: Left Atrial; LDH: Lactic Dehydrogenase; LVESVI: Left Ventricular End-Systolic Volume Index; LVEDVI: Left Ventricular End-Diastolic Volume Index; EF: Ejection Fraction; RA: Right Atrial; RV: Right Ventricular; Right Ventricular Wall Thickness; PA: Pulmonary Artery; PASP: Pulmonary Arterial Systolic Pressure; PICC: Peripherally Inserted Central Catheter

### Risk factors associated with PE among AE-COPD patients

Multivariable logistic regression showed that the Han nationality had a 43.4% (OR = 0.566, 95% CI 0.363, 0.883) lower probability of developing PE compared to other ethnic minorities. For every unit increase in neutral count, the patient’s risk of PE increased by 11.2% (OR = 1.112, 95% CI 1.037, 1.191). D-dimer > 1 mg/L (OR = 1.725, 95% CI 1.108, 2.686) and AST > 40 U/L (OR = 2.310, 95% CI 1.384, 3.856) showed a risk effect on PE. Furthermore, chest pain (OR = 2.121, 95% CI 1.229, 3.662), cardiac insufficiency or respiratory failure (OR = 7.451, 95% CI 4.691, 11.833), Padua score ≥ 4 (OR = 3.542, 95% CI 2.247, 5.583), and DVT (OR = 11.067, 95% CI 3.809, 32.156) were also associated higher probability of PE (Table 3). Additionally, the C-index of this model was 0.84 (Figure S2).

**Table 3:** Related factors of pulmonary embolism in patients with AE-COPD based on multivariable logistic regression

Variables	OR	95%CI	*P*-value
Nationality (Han vs. minority)	0.566	0.363, 0.883	**0.012**
Neutrophil count (per 1 × 10^9^)	1.112	1.037, 1.191	**0.003**
D-dimer (> 1 mg/L vs. ≤1 mg/L)	1.725	1.108, 2.686	**0.016**
AST (> 40 U/L vs. ≤40 U/L)	2.310	1.384, 3.856	**0.001**
Chest pain (with vs. without)	2.121	1.229, 3.662	**0.007**
Hypertension (with vs. without)	0.675	0.438, 1.039	0.074
Cardiac insufficiency or respiratory failure (with vs. without)	7.451	4.691, 11.833	**< 0.001**
Stroke within 3 months (with vs. without)	1.983	0.672, 5.856	0.215
Padua score (≥ 4 vs. 0–3)	3.542	2.247, 5.583	**< 0.001**
DVT (with vs. without)	11.067	3.809, 32.156	**< 0.001**
Lower limb pain (with vs. without)	1.935	0.447, 7.849	0.356

The C-statistic of this model was 0.84

Abbreviation: AECOPD: Acute Exacerbation of Chronic Obstructive Pulmonary Disease; AST: Aspartate Aminotransferase; DVT: Deep Vein Thrombosis

## Discussion

Currently, there is limited information on the prevalence and association between PE and AE-COPD in high-altitude areas. This study has the widest sample size to date, providing evidence to support their relationship. Initially, our research results showed that the incidence of PE was significantly higher compared to studies conducted in low altitude areas, so it is necessary to conduct further research and validation in populations living in high-altitude areas [25]. Additionally, our current study found the associations between PE and elevated Padua score, the presence of deep venous thrombosis, increased neutrophil count, chest pain, cardiac insufficiency or respiratory failure, higher levels of AST, and an elevated D-dimer level in hospitalized patients experiencing AE-COPD.

Many studies have shown that high-altitude areas are considered a potential risk factor for VTE, as challenging environmental conditions may enhance venous stasis and promote the formation of a prethrombotic environment [26–29]. The increase in PE prevalence among AE-COPD patients at high altitudes can likely be attributed to several factors. Firstly, living in high-altitude areas can expose individuals to cooler temperatures, reduced humidity, strong solar radiation, and low-pressure hypoxia conditions, which may promote physiological adaptation, such as changes in lung capacity or diffusion capacity [7]. The high-altitude adaptation can lead to increased lung ventilation, which in turn leads to blood concentration [30]. At the same time, an increase in diffusion volume in COPD patients can also lead to an increase in blood viscosity [31]. Consequently, this may increase the risk of thromboembolism in patients with COPD, contributing to factors such as systemic inflammation, hypoxemia, oxidative stress, endothelial dysfunction, and a prothrombotic state [32]. Secondly, research has indicated that environmental factors such as hypoxia, dehydration, hemoconcentration, reduced temperature, and venous stasis resulting from severe weather conditions at high altitudes can trigger a state of hypercoagulability, amplifying the occurrence of thromboembolic events [10, 33, 34]. Thirdly, high altitude triggers compensatory proliferation of red blood cells, increases blood viscosity, accelerates the consumption of coagulation factors, and leads to prolonged prothrombin time and partial activation time of thrombin. Moreover, numerous studies have established an elevation in fibrinogen levels at high altitudes, positively correlating with plasminogen activator inhibitor-1 (PAI-1) levels, and PAI-1 is an inhibitor of plasminogen activator, reducing fibrinolytic activity and further promoting the formation of thrombosis [10, 35–37].

Multivariable analysis showed patients in plateau regions with higher Padua scores, DVT, chest pain, cardiac insufficiency or respiratory failure, higher neutrophil count, AST, and D-dimer were more likely to develop PE. The results indicated a complex origin or the correlation of PE with factors such as advanced age, greater chronicity and severity of illness, stasis, infection, and an elevated inflammatory and coagulopathic state in hospitalized patients experiencing AE-COPD at high altitudes. Hence, our conjecture is that the inflammatory state and activation of coagulation mechanisms could contribute to the promotion of pulmonary embolism under hypoxic conditions at high altitudes [38]. Our results imply that residing at high altitudes could pose an additional risk for PE, potentially contributing to its increased prevalence. Potential mechanisms linking PE and high altitude may involve the release of thrombogenic cytokines induced by high-altitude conditions. However, further investigation is essential to elucidate the pathophysiology of PE in plateau regions.

Clinical trial findings suggest that the prevention [39, 40] and reduction of VTE duration [41, 42] can improve the clinical outcomes of critically ill patients. Our data indicates a potential protective effect of prophylaxis against PE in this higher-risk cohort. This underscores the importance of recognizing and reinforcing thromboprophylaxis strategies for AE-COPD, which may involve considering a moderate increase in the dosage of anticoagulant drugs and enhancing the utilization of physical prophylaxis.

While the findings were anticipated, given our patient population comprised individuals with AE-COPD at elevated risk for PE in plateau regions, our data prompts consideration of screening for PE, risk stratification, and potential prophylactic measures to enhance outcomes in hospitalized AE-COPD patients. Additionally, as PE lacks specific clinical manifestations and may mimic other respiratory diseases in the early stages, the possibility of a combined presentation cannot be ruled out. These complexities make PE diagnosis challenging. Therefore, heightened attention is warranted for high-risk PE groups within the AE-COPD population, emphasizing the importance of PE prophylaxis.

We have to acknowledge that there were some limitations in this study. Primarily, our study is limited by being a single-center prospective investigation, making it challenging to eliminate the possibility of selected bias. Secondly, the constrained availability of CTPA examinations due to the acute exacerbation condition in COPD patients led to a substantial underestimation of the prevalence of PE. Consequently, prospective multi-center studies with larger sample sizes may be essential in the future to validate the outcomes observed in our current study.

## Conclusions

The prevalence of PE was higher, and the risk factors for PE are a higher Padua score, the occurrence of deep venous thrombosis, higher neutrophil count, chest pain, cardiac insufficiency or respiratory failure, higher levels of AST, and a higher level of D-dimer among AE-COPD patients. The analysis of AE-COPD may help to provide more accurate screening for PE and lead to corresponding measures to improve the clinical outcomes of patients with AE-COPD in plateau regions.

### Electronic supplementary material

Below is the link to the electronic supplementary material.

Supplementary Material 1

## Data Availability

No datasets were generated or analysed during the current study.

## References

[1] Global regional. and national deaths, prevalence, disability-adjusted life years, and years lived with disability for chronic obstructive pulmonary disease and asthma, 1990–2015: a systematic analysis for the Global Burden of Disease Study 2015. The Lancet Respiratory medicine. Sep 2017;5(9):691–706. 10.1016/s2213-2600(17)30293-x. 10.1016/S2213-2600(17)30293-X PMC5573769 28822787

[2] Anees Ur R, Ahmad Hassali MA, Muhammad SA (2020). The economic burden of chronic obstructive pulmonary disease (COPD) in the USA, Europe, and Asia: results from a systematic review of the literature. Expert Rev Pharmacoeconomics Outcomes Res Dec.

[3] Zhu B, Wang Y, Ming J, Chen W, Zhang L (2018). Disease burden of COPD in China: a systematic review. Int J Chronic Obstr Pulm Dis.

[4] Wang C, Xu J, Yang L (2018). Prevalence and risk factors of chronic obstructive pulmonary disease in China (the China Pulmonary Health [CPH] study): a national cross-sectional study. Lancet (London England) Apr.

[5] Wedzicha JA, Seemungal TA. COPD exacerbations: defining their cause and prevention. Lancet. Sep 1. 2007;370(9589):786–96. 10.1016/s0140-6736(07)61382-8. 10.1016/S0140-6736(07)61382-8 PMC7134993 17765528

[6] Husebø GR, Gabazza EC, D’Alessandro Gabazza C, Respirology, Carlton (2021). Vic) Apr.

[7] Burtscher M (2014). Effects of living at higher altitudes on mortality: a narrative review. Aging Disease Aug.

[8] Białas AJ, Kornicki K, Ciebiada M (2018). Monocyte to large platelet ratio as a diagnostic tool for pulmonary embolism in patients with acute exacerbation of chronic obstructive pulmonary disease. Polish archives of internal medicine. Jan.

[9] Cao YQ, Dong LX, Cao J (2018). Pulmonary embolism in patients with Acute Exacerbation of Chronic Obstructive Pulmonary Disease. Chinese medical journal. Jul.

[10] Gupta N, Ashraf MZ. Mar. Exposure to high altitude: a risk factor for venous thromboembolism? Seminars in thrombosis and hemostasis. 2012;38(2):156–63. 10.1055/s-0032-1301413. 10.1055/s-0032-1301413 22422330

[11] Lippi G, Franchini M, Guidi GC. Dec. Prohibition of artificial hypoxic environments in sports: health risks rather than ethics. Applied physiology, nutrition, and metabolism = Physiologie appliquee, nutrition et metabolisme. 2007;32(6):1206-7; discussion 1208-9. 10.1139/h07-088. 10.1139/H07-088 18059596

[12] Toff WD, Jones CI, Ford I (2006). Effect of hypobaric hypoxia, simulating conditions during long-haul air travel, on coagulation, fibrinolysis, platelet function, and endothelial activation. Jama May.

[13] Anand AC, Jha SK, Saha A, Sharma V, Adya CM (2001). Thrombosis as a complication of extended stay at high altitude. Natl Med J India Jul-Aug.

[14] Ashraf HM, Javed A, Ashraf S (2006). Pulmonary embolism at high altitude and hyperhomocysteinemia. J Coll Physicians Surgeons–Pakistan: JCPSP Jan.

[15] Anand AC, Saha A, Kumar R, Sharma V, Jha SK (2000). Portal system thrombosis: a new dimension of high altitude illnesses. Trop Gastroenterol: Official J Dig Dis Foundation Oct-Dec.

[16] Zavanone C, Panebianco M, Yger M (2017). Cerebral venous thrombosis at high altitude: a systematic review. Revue Neurologique Apr.

[17] Abbasi AA, Harrop NS (2005). An unusual swelling in the neck. Emerg Med J: EMJ Sep.

[18] Pandey P, Lohani B, Murphy H (2016). Pulmonary embolism masquerading as high Altitude Pulmonary Edema at High Altitude. High Altitude Med Biology Dec.

[19] Yanamandra U, Boddu R, Pramanik S (2020). Prevalence and clinical characteristics of Post-thrombotic Syndrome in High-Altitude-Induced Deep Vein thrombosis: experience of a single Tertiary Care Center from Real-World Settings. High Altitude Med Biology Dec.

[20] Jha SK, Anand AC, Sharma V, Kumar N, Adya CM (2002). Stroke at high altitude: Indian experience. High Altitude Med Biology Spring.

[21] Smallman DP, McBratney CM, Olsen CH, Slogic KM, Henderson CJ. Feb. Quantification of the 5-year incidence of thromboembolic events in U.S. Air Force Academy cadets in comparison to the U.S. Naval and Military Academies. Military medicine. 2011;176(2):209–13. 10.7205/milmed-d-10-00144. 10.7205/milmed-d-10-00144 21366086

[22] Halpin DMG, Criner GJ, Papi A, Prevention of Chronic Obstructive Lung Disease. The 2020 GOLD Science Committee Report on COVID-19 and Chronic Obstructive Pulmonary Disease. Am J Respir Crit Care Med. Jan 1. Global Initiative for the Diagnosis, Management, and. 2021;203(1):24–36. 10.1164/rccm.202009-3533SO. 10.1164/rccm.202009-3533SO PMC7781116 33146552

[23] Lee JW, Cha SI, Jung CY (2009). Clinical course of pulmonary embolism in lung cancer patients. Respir Int Rev Thorac Dis.

[24] Cham MD, Yankelevitz DF, Shaham D (2000). Deep venous thrombosis: detection by using indirect CT venography. The pulmonary angiography-indirect CT Venography Cooperative Group. Radiol Sep.

[25] Liu X, Jiao X, Gong X (2023). Prevalence, risk factor and clinical characteristics of venous Thrombus embolism in patients with Acute Exacerbation of COPD: a prospective Multicenter Study. Int J Chronic Obstr Pulm Dis.

[26] Jha PK, Vijay A, Prabhakar A (2021). Transcriptome profiling reveals the endogenous sponging role of LINC00659 and UST-AS1 in high-Altitude Induced thrombosis. Thromb Haemostasis Nov.

[27] Srivastava S, Kumari B, Garg I et al. May. Targeted gene expression study using TaqMan low density array to gain insights into venous thrombo-embolism (VTE) pathogenesis at high altitude. Blood cells, molecules & diseases. 2020;82:102421. 10.1016/j.bcmd.2020.102421. 10.1016/j.bcmd.2020.102421 32171843

[28] Shang C, Wuren T, Ga Q (2020). The human platelet transcriptome and proteome is altered and pro-thrombotic functional responses are increased during prolonged hypoxia exposure at high altitude. Platelets.

[29] Gupta N, Sahu A, Prabhakar A (2017). Activation of NLRP3 inflammasome complex potentiates venous thrombosis in response to hypoxia. Proc Natl Acad Sci United States Am May 2.

[30] Berger MM, Luks AM. Oct. High Altitude. Semin Respir Crit Care Med. 2023;44(5):681–95. 10.1055/s-0043-1770063. 10.1055/s-0043-1770063 37816346

[31] Shi F, Cao J, Zhou D (2024). Revealing the clinical effect and biological mechanism of acupuncture in COPD: a review. Biomed Pharmacother Jan.

[32] Mejza F, Lamprecht B, Niżankowska-Mogilnicka E, Undas A (2015). Arterial and venous thromboembolism in chronic obstructive pulmonary disease: from pathogenic mechanisms to prevention and treatment. Pneumonologia i Alergologia Polska.

[33] Rocke AS, Paterson GG, Barber MT et al. Jan. Thromboelastometry and platelet function during acclimatization to high Altitude. Thrombosis and haemostasis. 2018;118(1):63–71. 10.1160/th17-02-0138. 10.1160/TH17-02-0138 PMC6260116 29304526

[34] Li M, Tang X, Liao Z (2022). Hypoxia and low temperature upregulate transferrin to induce hypercoagulability at high altitude. Blood Nov.

[35] Wang Z, Liu H, Dou M (2017). The quality changes in fresh frozen plasma of the blood donors at high altitude. PLoS ONE.

[36] Vij AG (2009). Effect of prolonged stay at high altitude on platelet aggregation and fibrinogen levels. Platelets Sep.

[37] Kotwal J, Apte CV, Kotwal A, Mukherjee B, Jayaram J (2007). High altitude: a hypercoagulable state: results of a prospective cohort study. Thromb Res.

[38] Beristain-Covarrubias N, Perez-Toledo M, Thomas MR, Henderson IR, Watson SP, Cunningham AF (2019). Understanding infection-Induced thrombosis: lessons learned from animal models. Front Immunol.

[39] Leizorovicz A, Cohen AT, Turpie AG, Olsson CG, Vaitkus PT, Goldhaber SZ (2004). Randomized, placebo-controlled trial of dalteparin for the prevention of venous thromboembolism in acutely ill medical patients. Circulation Aug.

[40] Samama MM, Cohen AT, Darmon JY (1999). A comparison of enoxaparin with placebo for the prevention of venous thromboembolism in acutely ill medical patients. Prophylaxis in Medical patients with Enoxaparin Study Group. New Engl J Med Sep.

[41] Cohen AT, Harrington RA, Goldhaber SZ (2016). Extended thromboprophylaxis with Betrixaban in acutely Ill medical patients. The New England journal of medicine. Aug 11.

[42] Cohen AT, Spiro TE, Büller HR (2013). Rivaroxaban for thromboprophylaxis in acutely ill medical patients. New Engl J Med Feb.

