# Supplementary material for: Prevalence, risk factors, and clinical characteristics of pulmonary embolism in patients with acute exacerbation of COPD in Plateau regions: a prospective cohort study
Source: BMC Pulm Med. 2024 Feb 27;24:102. doi: 10.1186/s12890-024-02915-z (PMC10900782; doi:10.1186/s12890-024-02915-z)
Supplement: Supplementary file 1 — Supplementary Material 1 [file 12890_2024_2915_MOESM1_ESM.docx]

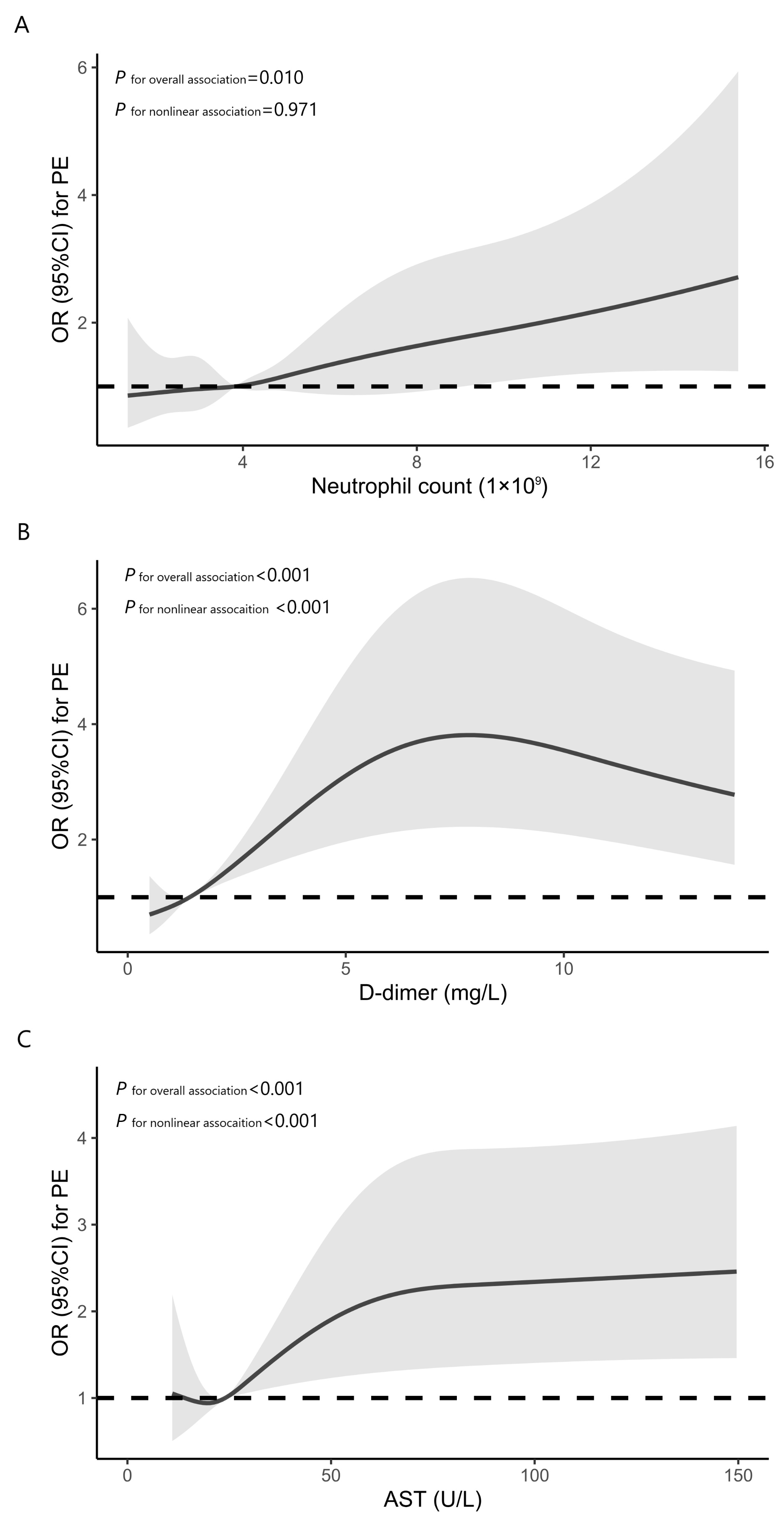
Supplementary Materials

Figure S1 Restrictive cubic spline plot of the association between neutrophil count, D-dimer, AST, and PE (A neutrophil count, B D-dimer, C AST)


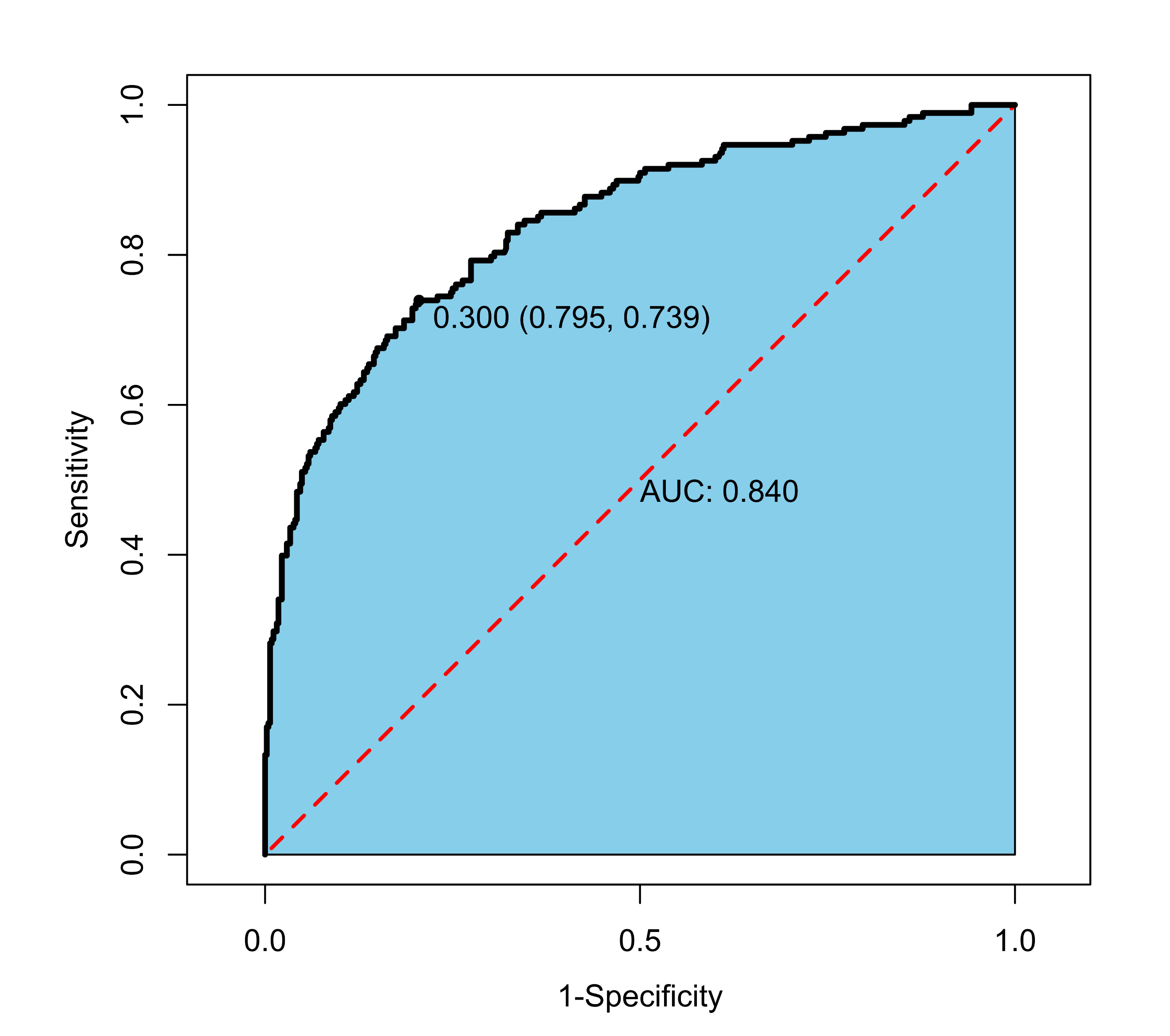


Figure S2 Receiver operating characteristic curve of multivariable logistic regression
